# The Epidemiological, Clinical, and Laboratory Features of Sporadic Creutzfeldt-Jakob Disease Patients in China: Surveillance Data from 2006 to 2010

**DOI:** 10.1371/journal.pone.0024231

**Published:** 2011-08-31

**Authors:** Chen Gao, Qi Shi, Chan Tian, Cao Chen, Jun Han, Wei Zhou, Bao-Yun Zhang, Hui-Ying Jiang, Jin Zhang, Xiao-Ping Dong

**Affiliations:** State Key Laboratory for Infectious Disease Prevention and Control, National Institute for Viral Disease Control and Prevention, Chinese Center for Disease Control and Prevention, Beijing, People's Republic of China; University of Melbourne, Australia

## Abstract

**Background:**

Creutzfeldt-Jakob disease (CJD) is a rare, rapidly progressive fatal central nervous system disorder, which consists of three main catalogues: sporadic, familial, and iatrogenic CJD.

**Methodology/Principal Findings:**

In China, the surveillance for CJD started in 2006, covering 12 provincial Centers for Disease Control and Prevention (CDCs) and 15 hospitals. From 2006 to 2010, 624 suspected patients were referred to China CJD surveillance. The epidemiological, clinical and laboratory features of sporadic CJD (sCJD) were analysed. Both groups of probable and possible sCJD showed highest incidences in the population of 60 to 69 year-olds. The most common presenting symptoms were progressive dementia and mental-related symptoms (neurological symptoms including sleeping turbulence, depression, anxiety and stress). Among the four main clinical manifestations, myoclonus was more frequently observed in the probable sCJD patients. About 2/3 of probable sCJD cases showed positive 14-3-3 in CSF and/or periodic sharp wave complexes (PSWC) in electroencephalography (EEG). The presence of myoclonus was significantly closely related with the appearance of PSWC in EEG. Polymorphisms of codon 129 in *PRNP* of the notified cases revealed a highly predominant M129M genotype in Han Chinese. Among 23 genetic human prion diseases, ten were D178N/M129M Fatal familial insomnia (FFI) and five were T188K genetic CJD (gCJD), possibly indicating a special distribution of gCJD-related mutations in Han Chinese.

**Conclusion:**

From the period of 2006 to 2010, 261 patients were diagnosed as sCJD and 23 patients were diagnosed as genetic human prion diseases in China. The epidemiological, clinical and laboratory analysis data were consistent with the characteristics of sporadic CJD, which provide insight into the features of CJD in China.

## Introduction

Creutzfeldt-Jakob disease (CJD) is a rare, rapidly progressive fatal central nervous system (CNS) disorder. It consists of three main catalogues: sporadic, familial and iatrogenic CJD. The reasons of sporadic CJD are still unclear. Genetic human prion disease is associated with a range of mutations in the prion protein gene (*PRNP*) on Chromosome 20 [1], which include genetic or familial CJD, as well as other genetic human transmissible spongiform encephalopathies (TSE), such as Gerstmann-Sträussler-Scheinker disease (GSS) and fatal familial insomnia (FFI). Iatrogenic CJD is acquired by various iatrogenic procedures, such as treatment with human pituitary growth hormones, dura mater and cornea grafts, deep brain electrodes and neurosurgery [2]. Since 1996, a new acquired human CJD, variant CJD (vCJD), has been recognized worldwide, which is confirmed to link to bovine spongiform encephalopathy (BSE) [3]. Up to now, 221 vCJD cases have been described, including one Japanese case.

Based on the current diagnostic criteria for sporadic CJD (sCJD) issued by WHO, the possible diagnosis of sCJD is based on the clinical manifestations, that is progressive dementia plus at least two out of four following signs: myoclonus, visual or cerebellar disturbance, pyramidal or extrapyramidal dysfunction and akinetic mutism. The probable diagnosis of sCJD needs further clinical data, such as typical changes in EEG and MRI, or laboratory data, such as a positive 14-3-3 protein test in cerebrospinal fluid (CSF). An absolutely definite diagnosis of any form of CJD usually requires either pathological or pathogenic examination of brain tissues.

As the great impact of the outbreak of BSE and the emerging cases of vCJD on public health, many countries or regions, particularly European countries, have started or restarted the surveillance programme for CJD. In 2006, a surveillance program for CJD was conducted in China, which was supported by Chinese Center for Disease Control and Prevention (CCDC). Up to the end of 2010, 284 various CJD and human TSE cases were identified and diagnosed. In this paper, we comparably describe the epidemical, clinical and laboratory features of Chinese CJD patients based on this surveillance.

## Materials and Methods

### Ethics statement

Usage of the human clinical samples in this study, which was one of the important clinical materials in China CJD Surveillance System, has been approved by the Ethical Committee of National Institute for Viral Disease Prevention and Control, China CDC. All signed informed consents have been collected and stored by the China CJD Surveillance Center.

### Constitution of China CJD surveillance

China CJD surveillance started formally in 2006, under the framework of the national communicable surveillance network of the CCDC. It consisted of 10 provincial CDCs and 11 hospitals, distributing in Beijing, Shanghai, Tianjin, Chongqing, Jilin, Shaanxi, Hubei, Guangdong, Guizhou and Anhui. In 2008, two provincial CDCs and three hospitals in Henan and Xinjiang joined in the network. The surveillance program was approved by the Ethical Review Committee of CCDC. Based the surveillance documents, the patients who have symptoms in accordance with the diagnosis criteria for at least possible sCJD or other subtypes of human prion diseases, or any case that is believed by the physicians to be necessary to exclude the possibility of CJD can be referred as a “suspected CJD case” to the surveillance system. The clinical data and specimens of the suspected patients were collected by the clinicians from hospitals, while the epidemiological data were collected by the staff of provincial CDCs. The collected data and samples were referred to the national reference laboratory for human prion disease, CCDC, for laboratory tests and final diagnosis.

### Collections of clinical and epidemiological data

The clinical and epidemiological data of suspected CJD patient were collected with the designed questionnaires. The clinical data included the general information, main clinical manifestations, the presenting symptoms and clinical examinations (for example, CT, MRI, EEG and routine CSF biochemistry). The epidemiological data included inhabitancy, family history, anamnesis (surgical or neurosurgical history, organ transplantation, blood donation and transfusion, use of extracts of pituitary or other blood products), and profession (especially medical staff, veterinarians and butchers).

### Laboratory tests

The specimens of blood, CSF and brain tissues of the suspected patient were collected by the sentinel hospital. All samples were transported to the national reference laboratory for human prion disease in CCDC. The peripheral blood leukocytes were used for sequencing analysis of *PRNP* and polymorphism of codon 129, with an automatic genetic analyzer (ABI3130XL). CSF samples were obtained by routine lumbar puncture and analysed by Western immunoblot to detect the 14-3-3 protein. Brain tissues obtained from autopsy or biopsy were applied into neuropathologic assays and/or PrP^Sc^ detections with immunohistochemistry and/or Western blot. The standard operation procedure (SOP) of each test was well documented in the CJD surveillance program, which was described previously [4].

### Case definition

The suspected CJD cases reported from CJD surveillance were diagnosed and subtyped based on the diagnostic criteria issued by CCDC, which was constituted based on the diagnostic criteria for CJD issued by WHO. Since 2010, high signal in caudate/putamen on MRI brain scan has been added into the criteria according to the renewed version issued by WHO [5]. The final diagnosis was made by an expert board including neurologists, neuropathologists, epidemiologists and laboratory staff.

### Statistical analysis

All statistical analysis were performed with the Chi-square test using the SPSS 11.5 statistical software programme and statistical significance corresponds to a *p* value<0.05.

## Results

Between 2006 and 2010, 624 suspected CJD cases were referred to the China CJD surveillance program. The notified numbers of suspected CJD patients increased year by year, but without seasonal relativity. The resident sites of reported cases distributed widely in mainland of China, but most cases were reported from the hospitals in larger cities. Based on the diagnostic criteria for sCJD, 163 patients were classified as probable sCJD and 96 patients were diagnosed as possible sCJD. Only a few cases had brain biopsies, among them two cases were diagnosed as definite sCJD in term of positive presences of PrP^Sc^ in brain tissues with Western blots or immunohistochemistry. In addition, 23 genetic human prion diseases were diagnosed based on the genetic assays of *PRNP*, including ten FFI (D178N/M129M) cases, two GSS (P102L) cases, five T188K gCJD cases, two G114V gCJD cases, one E196K gCJD case, one E200K gCJD case, one R208H gCJD case and one gCJD cases with one extra-octarepeat insertion. Some of the genetic prion diseases have been already described as case report [6], [7], [8], [9].

### General sociodemographic features

**Figure 1:**
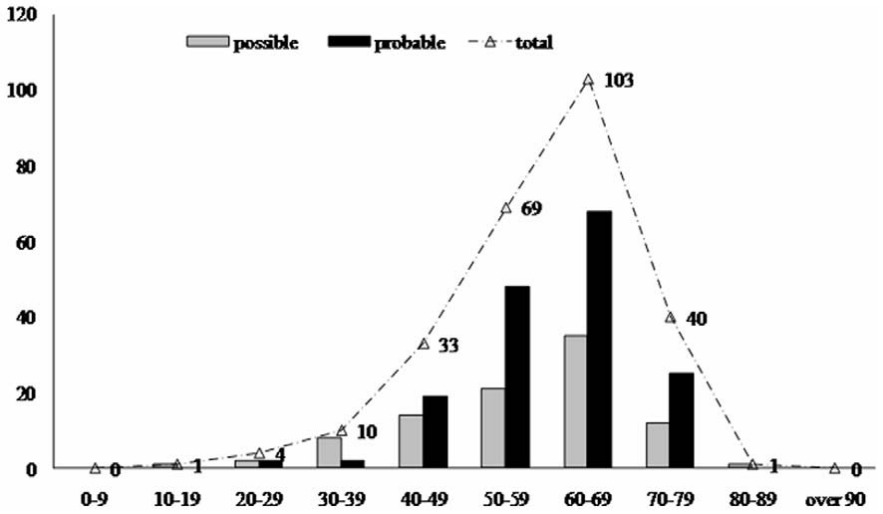
The age distribution of probable and possible sCJD cases. The black column indicates probable sCJD, the grey column indicates possible sCJD and the curve represents the total numbers.

**Figure 2:**
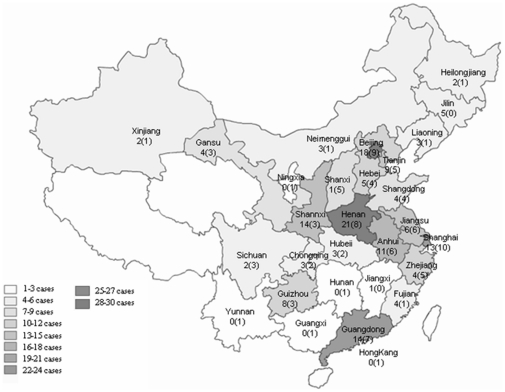
The geographic distribution of probable and possible sCJD cases based on the permanent residences. The numbers of probable and possible sCJD cases in each province were showed as X (X), respectively.

Out of 261 sCJD patients, 146 were males and 115 were females, with the gender ratio of 1.27∶1. The onset age of probable sCJD ranged from 21 to 82 y, with the median age of 61 y, while that of possible sCJD ranged from 18 to 80 y, with the median age of 60 y. Both groups of sCJD showed highest incidences in the population of 60 to 69 year-olds (Fig. 1). The patients distributed widely in term of their permanent resident sites (Fig. 2), 78.4% lived in the city and 21.6% in the countryside. The occupations and educational levels of sCJD patients varied considerably. Five cases were retired medical staff, but after careful investigation, we excluded the possibility of iatrogenic infection. All cases showed no linkage with their anamnesis. The onsets occurred all year round, without seasonal specificity.

### Clinical features

The presenting symptoms of the reported sCJD cases were multifarious. Progressive dementia was the most common presenting symptoms that were noted by 118 out of 259 probable and possible sCJD (45.6%) patients. Mental syndrome (including sleeping turbulence, depression, anxiety and stress) appeared in 61 patients (23.6%), followed by pyramidal or extrapyramidal dysfunction (17.0%), cerebellum syndrome (16.6%), slow progressive dementia (12.7%) and visual disturbance (10.0%). Analysis of the appearance percentages of the presenting clinical manifestations in probable and possible sCJD patients revealed similar patterns (Table 1). Only the percentages of slow progressive dementia that was persistent longer than one year in the group of possible sCJD were slightly higher than that in probable sCJD, but without statistical significance.

**Table 1:** The presenting symptoms of the probable and possible sCJD patients.

clinical manifestation	case number		
	probable	possible	*P*
progressive dementia	79 (48.5%)	39 (40.6%)	0.2219
mental syndrome^1^	40 (24.5%)	21 (21.9%)	0.6261
cerebellum syndrome	27 (16.6%)	16 (16.7%)	0.9830
pyramidal or extrapyramidal dysfunction	27 (16.6%)	17 (17.7%)	0.8132
visual disturbance	16 (9.8%)	10 (10.4%)	0.8768
slow progressive dementia^2^	17 (10.4%)	16 (16.7%)	0.1467
cerebral stroke	3 (1.8%)	1 (1.0%)	0.6152
dizziness	4 (2.5%)	1 (1.0%)	0.4259
sleeping disturbance	1 (0.8%)	1 (1.0%)	0.7043
lower limbs inability	2 (1.3%)	\	0.2768
speech disability	\	1 (1.0%)	0.1926
headache	2 (1.3%)	\	0.2768

1 including sleeping turbulence, depression, anxiety and stress etc.

2 when the dementia persists longer than one year progressively without other manifestations.

Almost all probable and possible sCJD patients displayed obviously progressive dementia in their clinical courses. Except for progressive dementia, the presence of four other clinical manifestations included in the diagnostic criteria for sCJD, myoclonus, visual or cerebella disturbance, pyramidal or extrapyramidal dysfunction and akinetic mutism, were compared between two groups. Pyramidal or extrapyramidal dysfunction was the most commonly observed sign in both probable and possible patients, which accounted 77.9% and 79.2%, respectively. Visual or cerebellar disturbances are noted comparably in probable (57.1%) and possible (58.3%) sCJD patients. Akinetic mutism appeared in 38.0% improbable and 30.2% possible patients. Myoclonus was more frequently observed in the group of probable sCJD, showing statistical differences (Table 2).

**Table 2:** The appearances of main clinical manifestations except progressive dementia in probable and possible sCJD patients.

	case number	myoclonus	visual or cerebellar disturbance	pyramidal or extrapyramidal dysfunction	akinetic mutism
Probable sCJD	163	121 (74.2%)	93 (57.1%)	127 (77.9%)	62 (38.0%)
Possible sCJD	96	54 (56.3%)	56 (58.3%)	76 (79.2%)	29 (30.2%)
*P*		0.0028	0.841	0.8134	0.2469

As sCJD has no special clinical manifestation, the appearances of symptoms and signs may vary considerably. Analysis of the frequencies of those four main manifestations in the groups of probable and possible sCJD showed the similar tendencies, that small portions of the patients had all four neurological manifestations. However, the number of patients who had three signs in the group of probable sCJD were markedly larger than that of possible sCJD (*P* = 0.0055). In contrast, a remarkably larger number of possible sCJD patients (68.8%) showed only two symptoms than that of probable sCJD ones (47.9%) (*P* = 0.0011, Table 3).

**Table 3:** The frequencies of other four clinical manifestations except progressive dementia in probable and possible sCJD patients.

	case number	having four clinical features	having three clinical features	having two clinical features
Probable sCJD	163	24 (14.7%)	61 (37.4%)	78 (47.9%)
Possible sCJD	96	10 (10.4%)	20 (20.8%)	66 (68.8%)
*P*		0.3224	0.0055	0.0011

### Laboratory features

**Figure 3:**
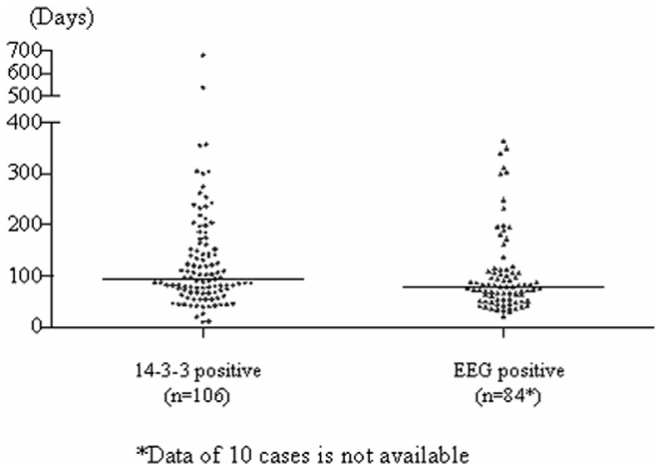
The distribution of the times positively detecting 14-3-3 in CSF (blue dot) and/or PSWC on EEG (green dot) after the onset in the probable sCJD patients. The black lines indicate the median data.

Appearances of 14-3-3 protein in CSF and typical PSWC on EEG were important indicators for probable sCJD. Most of the reported cases had only one examination of CSF 14-3-3 and/or EEG during their clinical course. Among 163 probable sCJD patients, 153 patients had CSF 14-3-3 tests and 69.3% (106) were positive, while 148 patients had EEG examinations and 63.5% (94) showed PSWC. Both 14-3-3 in CSF and PSWC on EEG could be detected in an extended span of time after onset of symptoms (Fig. 3), among them the earliest and latest data of CSF 14-3-3 were 10 and 680 (median: 95) days after onset, while those of PSWC in EEG were 20 and 340 (median: 78.5) days after onset. No significant difference in the identifying time of the abnormality was found between the groups of CSF 14-3-3 positive and EEG abnormality. In terms of the presence of four main clinical manifestations, more percentage of the patients with EEG abnormality showed myoclonus during their clinical courses than those with 14-3-3 positive (*P* = 0.0421), whereas other three manifestations distributed comparably in these two groups.

In all probable sCJD cases, 138 patients had both examinations of EEG and CSF 14-3-3 during their clinical course. 62 cases showed 14-3-3 positive in CSF and PSWC on EEG, 44 were only CSF 14-3-3 positive, 32 were only PSWC in EEG. The appearances of the four main clinical features were comparably analyzed among various groups. Table 4 showed that roughly 3/4 and 2/3 patients in each group had pyramidal or extrapyramidal dysfunction and visual or cerebellar disturbance, respectively. Myoclonus was apparently frequently observed in the patients with PSWC in EEG (*P* = 0.008) and in those with both CSF 14-3-3 and PSWC in EEG (*P* = 0.0076) (Table 4), showing an obviously EEG abnormality-related manner. Akinetic mutism seemed to be more frequent in the group of CSF 14-3-3 positive and PSWC in EEG, but without statistic difference.

**Table 4:** Comparison of the results of 14-3-3 and EEG with the appearances of clinical manifestations in 138 probable sCJD patients with two examinations.

	case number	myoclonus	visual or cerebellar disturbance	pyramidal or extrapyramidal dysfunction	akinetic mutism
14-3-3	44	24 (54.5%)	27 (61.4%)	35 (79.5%)	15 (34.1%)
EEG	32	29 (90.6%)*	18 (56.3%)	27 (84.4%)	11 (34.4%)
14-3-3+EEG	62	49 (79.0%)**	36 (58.1%)	46 (74.2%)	28 (45.2%)

*compared with the data of 14-3-3 positive, *P* = 0.0008.

**compared with the data of 14-3-3 positive, *P* = 0.0076.

Analysis of the frequencies of four main neurologic features among above three groups revealed that more percentages of the patients with both abnormalities in CSF 14-3-3 and PSWC on EEG had four and three symptoms during clinical courses, but without statistic difference compared with the patients with only one abnormality either in CSF 14-3-3 or on EEG (Table 5). However, significantly fewer patients with both abnormalities in CSF 14-3-3 and EGG had only two symptoms than those that were only CSF 14-3-3 positive (*P* = 0.006). No statistic difference in the frequencies of four main neurologic features was observed between the groups of only CSF 14-3-3 positive and only PSWC on EEG.

**Table 5:** The frequencies of other four clinical manifestations except progressive dementia in 138 probable sCJD patients with two examinations.

	case number	having four clinical features	having three clinical features	having two clinical features
14-3-3	44	5 (11.4%)	10 (22.7%)	29 (65.9%)
EEG	32	4 (12.5%)	12 (37.5%)	16 (50.0%)
14-3-3+EEG	62	13 (21.0%)	25 (40.3%)	24 (38.7%)*

*compared with the data of 14-3-3 positive, *P* = 0.006.

*PRNP* sequences of 560 notified suspected CJD patients were analyzed. 548 patients showed a methionine homozygous genotype at codon 129 (97.9%), whereas 12 were methionin/valine heterozygote (2.1%) and none was valine/valine homozygote, revealing a typical Han Chinese frequency [10]. Only one person showed the E219K polymorphism. Analysis of the codon 129 polymorphism in probable and possible sCJD identified similar features, showing a highly predominant M129M genotype (96.9% in probable and 99.0% in possible). 22 genetic human prion diseases were diagnosed during 2006 to 2010 based on *PRNP* sequencings, all of them being M129M homozygote. Interestingly, ten D178N/M129M FFI and five T188K gCJD cases were identified, which accounted for 43.5% and 21.7% of diagnosed genetic human prion diseases. It may indicate that the genotypes of D178N/M129M and T188K distribute relatively widely among Han Chinese.

## Discussion

In this report, we describe the clinical, epidemiological and laboratory features of 261 sCJD cases who are diagnosed under the framework of surveillance network in mainland of China from 2006 to 2010. These included 2 definite, 163 probable and 96 possible cases of sCJD. The general sociodemographic characteristics of Chinese sCJD cases are coincident well with those described elsewhere [4], without detectable linkage among the cases. A large number of sCJD patients display their clinical manifestations at the ages of 60 to 69 year-old. The patients distribute widely in whole country according to their permanent residence. Some regions have more cases, e.g. Beijing, Shanghai and a couple of eastern provinces, but this may reflect merely a good knowledge of CJD among the local clinician and relatively developed medical service, instead of the actual morbidity. More male Chinese sCJD cases have been detected with a gender ratio of 126.9∶100, which is higher than the average gender ratio (106.3∶100) in mainland of China. The sCJD patients have a wide range of professions.

Regardless of probable or possible sCJD, progressive dementia is inevitably notified during the progression of disease [11]. The presenting symptoms of the probable and possible sCJD patients are quite similar, in which progressive dementia is most detectable, followed by mental syndrome, cerebellum syndrome and pyramidal or extrapyramidal dysfunction. Along with the progression of the disease, other neurologic symptoms have been gradually observed. Although there is no special difference in term of the presence of neurologic symptoms between probable and possible sCJD, remarkably more percentages of probable sCJD patients have three main clinical features than that of possible sCJD. Moreover, among the four main clinical manifestations, myoclonus is more frequently released in the probable sCJD patients. It may highlight a more severe and extended neuropathologic damage in probable sCJD patients.

As the important diagnostic markers for sCJD, the data of CSF 14-3-3 Western blot and EEG in notified cases have been collected through CJD surveillance network. Both 14-3-3 in CSF and PSWC on EEG can be detected in an extended range during clinical courses of the probable sCJD patients, among them CSF 14-3-3 seems to be detectable in a more extended range. Two patients with relatively long clinical courses more than 500 days are CSF 14-3-3 positive, but negative in EEG. Although probable sCJD patients show high ratios of both CSF 14-3-3 positive and PSWC in EEG, only roughly approximately 45% of patients who have undergone those two examinations are positive for both. It emphasizes again the irreplaceable roles of CSF 14-3-3 and PSWC on EEG in diagnosis of sCJD. From our data, it seems that the presence of myoclonus is more closely related with the appearance of PSWC on EEG.

All 560 notified cases that have performed *PRNP* analysis are Han Chinese. Similar to that previously reported [4], M129M are highly predominant either in notified patients or sCJD patients. The percentage of M129M in sCJD patients is slightly higher than that in normal Han Chinese [10], possibly indicating a CJD-related sensitivity. 23 genetic human prion diseases have been identified, which occupies 7.8% of all diagnosed human prion diseases in the past five years. Surprisingly, ten are D178N/M129M FFI and five are T188K gCJD, which accounting for the majority of the reported genetic human prion diseases. This differs from the distribution of genetic human TSE in Caucasian populations [12], [13], possibly suggesting a special *PRNP* patterns in Han Chinese. Besides FFI cases, almost all other human TSE are misdiagnosed as sCJD until *PRNP* gene sequencing. It implies again that sequence analysis of *PRNP* is an indispensable methodology for detection of gCJD, especially for the patients without detectable family histories.

The China CJD surveillance network is a hospital-based surveillance that contains 15 large hospitals in 12 cities. Most notified cases drive from the sentinel hospitals, except Beijing and Shanghai where the cases are reported from many different hospitals. It possibly reflects merely the good knowledge of CJD and high level of medical service in Beijing and Shanghai. CJD is not a notifiable disease in China. Even in the CJD surveillance covered cities, only 1 to 2 hospitals are involved in the surveillance activities. Hence, it is still too early to define the CJD morbidity and mortality based on population-size in China. An extremely low percentage of postmortems performed in the Chinese population makes it difficult to definitely diagnose CJD. Comprehensive understanding of the other features of CJD, besides its neuropathology, shows special benefits for Chinese neurologists and public health staff. Nevertheless, our five-year CJD surveillance mission not only provides the meaningful CJD data in China, but also makes it possible to perform the population-size based CJD surveillance in some special regions in the near future, such as Beijing and Shanghai.
